# Cerebrospinal fluid leakage after intradural spinal surgery in children

**DOI:** 10.1007/s00381-022-05797-w

**Published:** 2023-02-15

**Authors:** Emma M. H. Slot, Tristan P. C. van Doormaal, Kirsten M. van Baarsen, Niklaus Krayenbühl, Luca Regli, Menno R. Germans, Eelco W. Hoving

**Affiliations:** 1 Department of Neurology and Neurosurgery, University Medical Center Utrecht, Utrecht, The Netherlands; 2 Department of Translational Neuroscience, University Medical Center, Utrecht Brain Center, Utrecht University, Utrecht, The Netherlands; 3 Department of Neurosurgery, Clinical Neuroscience Center, University Hospital Zurich, University of Zurich, Zurich, Switzerland; 4 Department of Neuro-Oncology, Princess Máxima Center for Pediatric Oncology, Utrecht, The Netherlands; 5 Division of Pediatric Neurosurgery, University Children’s Hospital Zurich, University of Zurich, Zurich, Switzerland

**Keywords:** Cerebrospinal fluid leakage, Pediatrics, Infection, Spinal surgery, Spina bifida

## Abstract

**Purpose:**

This study aimed to establish the incidence of CSF leakage in children and associated complications after intradural spinal surgery in three tertiary neurosurgical referral centers and to describe the treatment strategies applied.

**Methods:**

Patients of 18 years or younger who underwent intradural spinal surgery between 2015 and 2021 in three tertiary neurosurgical referral centers were included. Patients who died or were lost to follow-up within six weeks after surgery were excluded. The primary outcome measure was CSF leakage within six weeks after surgery, defined as leakage of CSF through the skin. Secondary outcome measures included the presence of pseudomeningocele (PMC), meningitis, and surgical site infection (SSI).

**Results:**

We included a total of 75 procedures, representing 66 individual patients. The median age in this cohort was 5 (IQR = 0-13 years. CSF leakage occurred in 2.7% (2/75) of procedures. It occurred on days 3 and 21 after the index procedure, respectively. One patient was treated with a pressure bandage and an external lumbar drain on day 4 after diagnosis of the leak, and the other was treated with wound revision surgery on day 1 after the leak occurred. In total, 1 patient developed a PMC without a CSF leak which was treated with wound revision surgery. SSI occurred in 10.7%, which included both cases of CSF leak.

**Conclusions:**

CSF leakage after intradural spinal surgery in the pediatric population is relatively rare (2.7%). Nevertheless, the clinical consequences with respect to secondary complications such as infection and the necessity for invasive treatment are serious.

**Supplementary Information:**

The online version contains supplementary material available at 10.1007/s00381-022-05797-w.

## Introduction

Cerebrospinal fluid leakage is a potentially serious complication after intradural spinal surgery. The complications associated with CSF leakage include wound infection, meningitis, and CSF hypotension. CSF leakage may necessitate invasive treatment, such as surgical wound revision, and prolong hospitalization [1, 2]. In addition, it is associated with increased healthcare costs [1, 2].

Watertight closure is thought to be the most important step to prevent postoperative CSF leakage. Surgeons may make use of autologous or synthetic duraplasty material and may choose to use a sealant. Their efficacy in the prevention of CSF leakage, especially in the pediatric population, however, has not been studied.

There is variation in the definition of CSF leakage across different studies [3]. Some definitions of CSF leakage may include both incisional leakage and pseudomeningocele (PMC) [3]. Yet, incisional CSF leakage is defined as leakage of CSF through the skin and PMC is a collection of CSF under the skin [4]. PMC is often self-limiting [4]. Therefore, in the current study, we will define CSF leakage as incisional CSF leakage and consider PMC separately. Furthermore, reports on CSF leakage after intradural spinal surgery based on recent data are limited and mostly include a specific indication only [5–11].

This study aims to establish the risk of CSF leakage and associated complications after intradural spinal surgery in three tertiary neurosurgical referral centers between 2015 and 2021 and to describe the treatment strategies applied. The results will be compared to the existing literature.

Evaluation of the up-to-date risk of CSF leakage and associated complications in a multicenter setting may serve as a benchmark and assist in counseling of future patients and parents.

## Methods

### Study design

This is a multicenter retrospective cohort study of all consecutive pediatric patients in the Wilhelmina Children’s Hospital and Princess Máxima Center for Pediatric Oncology in Utrecht, the Netherlands, and the University Children’s and University Hospital in Zurich, Switzerland. This study was approved by the institutional research boards and the local ethics committee and conducted according to the STrengthening the Reporting of OBservational studies in Epidemiology guidelines [12].

### Study population

All patients of 18 years or younger who underwent intradural spinal surgery between January 1st, 2015, and June 30th, 2021, with a surgical report available were included. Patients who died within 6 weeks after surgery, were lost to follow-up, or had reoperation within 6 weeks for other reasons than CSF leakage treatment were excluded. Inclusion and exclusion criteria are presented in Online resource 1.

### Data collection

Procedures eligible for inclusion were screened based on a prospectively collected database or operation codes in the electronic patient records. Data was collected from the following sources: medical notes, medical imaging, and surgical reports between 6 weeks prior to surgery and 6 weeks after surgery.

Surgical characteristics collected were indication of surgery (tumor, vascular, developmental defect, trauma, infection), emergency surgery (yes/no), reoperation (defined as any consecutive surgery at the same anatomical location), the use of products with the intention to seal the dura (yes/no), duraplasty use (yes/no), attempt to watertight closure (yes/no), suture method used (continuous or standing), and suture material used.

The following patient data were collected: age at the time of surgery, steroid use for multiple consecutive days directly before or after surgery, diagnosis of hydrocephalus based on medical imaging or medical notes, perioperative CSF diversion surgery (external ventricular drainage (EVD), external lumbar drainage (ELD), ventriculoperitoneal (VP)-shunt, or endoscopic third ventriculostomy (ETV)), perioperative chemotherapy, and perioperative radiotherapy. Patients who used steroids directly before or after surgery, underwent perioperative chemotherapy, or were diagnosed with the auto-immune disease were classified as immunocompromised.

Data items related to the clinical course included the total number of admission days, the number of days in the pediatric intensive care unit (PICU), and new neurological deficit after surgery (including worsening of existing deficit). If CSF leakage had occurred, the following additional characteristics were retrieved: the estimated number of extra admission days due to CSF leakage (estimated based on a treatment course for CSF leakage or readmission days), surgical wound revision because of CSF leakage, a CSF diverting procedure as a treatment for CSF leakage, the number of days of CSF diversion, a lumbar puncture to treat CSF leakage, extra suture placement, or pressure bandage because of CSF leakage, puncture of PMC.

The primary outcome measure of this study was CSF leakage within 6 weeks after surgery, defined as leakage of CSF through the skin (either confirmed with a beta-2-transferrin test or reported in the clinical notes). Secondary outcome measures were the presence of PMC (defined as a subcutaneous collection of CSF), surgical site infection (SSI) (diagnosis or suspicion of SSI based on either antibiotic treatment according to the clinical notes, or a positive wound culture), and meningitis (suspicion or diagnosis of meningitis based on either antibiotic treatment according to the clinical notes, or positive CSF culture) within 6 weeks after surgery.

The PubMed and EMBASE database were searched until September 12, 2022, for studies reporting the CSF leakage incidence after spinal surgery in children. Studies including only one specific indication for surgery and case reports were excluded. The following search terms were used: (“cerebrospinal fluid leak*” OR “CSF leak*”) AND (“pediatric” OR “peadiatric” OR “child*”) AND (“spine” OR “spinal”). Reference lists of included articles were manually searched for additional relevant studies.

### Statistical analyses

Baseline characteristics and incidence of secondary complications were presented as median with interquartile range (IQR) and compared between patients with CSF leakage and those without, using the Mann–Whitney U test for continuous variables and Fisher’s exact test for categorical variables. Variables with ≥ 5% missing data were not included into statistical analyses. The primary outcome measure is presented as the percentage of the total population. *P*-values < 0.05 were considered significant. Because of the high number of potential risk factors for CSF leakage, Bonferroni correction was applied with a baseline significance level of 5%. All analyses were performed in IBM SPSS, version 26.0 (IBM Corp., Armonk, N.Y., USA).

## Results

### Demographics

**Fig. 1:**
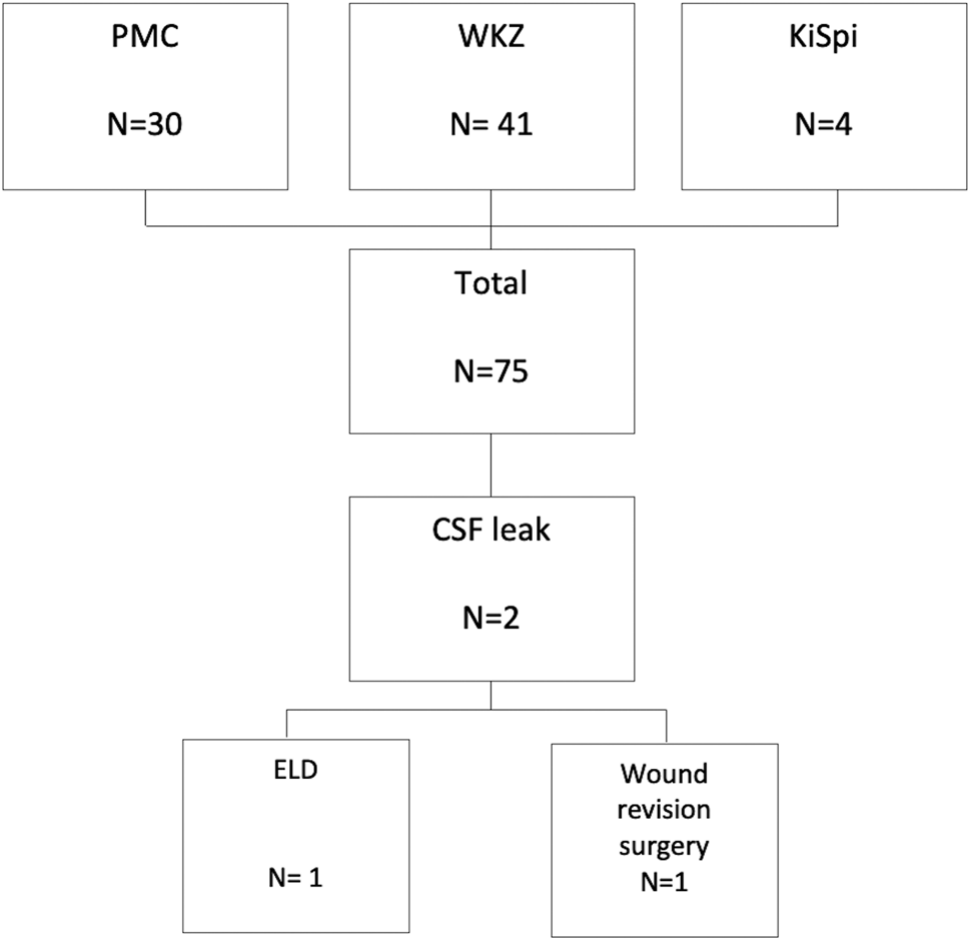
Flowchart of the study population. CSF, cerebrospinal fluid; ELD, external lumbar drainage; KiSpi, University Children’s Hospital Zurich; PMC, Princess Máxima Center for Pediatric Oncology; WKZ, Wilhelmina Children’s Hospital

We included 75 procedures, representing 66 individual patients (Fig. 1). The median age in this cohort was 5 (IQR = 0-13) years, with a male preponderancy (*n* = 43, 57.3%) (Table 1). The most frequent indication was tumor resection (*n* = 39), followed by developmental defects (*n* = 34) and vascular surgery (*n* = 2). Seventeen procedures were reoperations. Among the procedures for developmental defects were 11 myelomeningoceles, of which 1 had a preoperative leak (but no postoperative CSF leak).

**Table 1:** Study population characteristics

**Variable**	**Total (*N* = 75)**	**No CSF leakage (*N* = 73)**	**CSF leakage (*N* = 2)**	***P*-value**
*Patient characteristics*				
Age, years	5 (0-13)	5 (0.5-13)	3 (0-3)	0.450
Sex (female)	32 (42.7)	32 (43.8)	0 (0.0)	0.504
Immunocompromised	18 (24.0)	18 (24.7)	0 (0.0)	1.00
Radiation therapy	9 (12.0)	9 (12.3)	0 (0.0)	1.00
Chemotherapy	10 (13.3)	10 (13.7)	0 (0.0)	1.00
Hydrocephalus	7 (9.3)	7 (9.6)	0 (0.0)	1.00
CSF diversion	10 (13.3)	10 (13.7)	0 (0.0)	1.00
*Surgical characteristics*				
Indication				0.025
*Tumor*	*39 (52.0)*	*39 (53.4)*	*0 (0.0)*	
*Vascular*	*2 (2.7)*	*1 (1.4)*	*1 (50.0)*	
*Developmental defect*	*34 (45.3)*	*33 (45.2)*	*1 (50.0)*	
*Infection*	*0 (0.0)*	*0 (0.0)*	*0 (0.0)*	
*Trauma*	*0 (0.0)*	*0 (0.0)*	*0 (0.0)*	
Reoperation	18 (24.0)	18 (24.7)	0 (0.0)	1.00
Emergency surgery	10 (13.3)	9 (12.3)	1 (50.0)	0.250
Sealant use	50 (66.7)	49 (67.1)	1 (50.0)	1.00
Duraplasty use	4 (5.4)	4 (5.5)	0 (0.0)	1.00
*Autologous*	1 (1.4)	1 (1.4)	0 (0.0)	
*Non-autologous*	3 (4.1)	3 (4.1)	0 (0.0)	
Watertight dura closure	71 (94.7)	70 (95.9)	1 (50.0)	NP
Suture material used				NP
*PDS*	38 (50.7)	38 (52.1)	0 (0.0)	
*Vicryl*	30 (40.0)	29 (39.7)	1 (50.0)	
Method of suturing				NP
*Continuous*	52 (69.3)	51 (69.9)	1 (50.0)	
*Standing*	1 (1.3)	1 (1.4)	0 (0.0)	
*Clinical course characteristics*				
Hospital stay, days	6 (4-10)	6 (4-9)	14 (11-14)	0.072
PICU stay, days	0 (0-1)	0 (0-1)	0 (0-0)	0.398
New/increased neurological deficit	12 (16.0)	12 (16.4)	0 (0.0)	1.00

All values are reported as numbers with percentages between brackets for categorical values and median with interquartile range (IQR) between brackets for continuous variables. Percentage of data missing: duraplasty use: 1.8%, watertight dura closure 5.3%, suture material 9.3%, method of suturing 29.3%. No data were missing for the remaining variables. Fisher’s exact test was used for categorical variables and the Mann–Whitney U test for continuous variables

*CSF* cerebrospinal fluid, *N* number, *NP* not performed, *PDS* polydioxanone suture, *PICU* intensive care unit

In total, 10 patients had undergone CSF diversion surgery, of whom 5 preoperatively and 5 postoperatively. In total, 1 patient who underwent tumor resection surgery had undergone preoperative ETV and 4 patients had a VP-shunt, of whom one patient was treated for a tumor and 3 for developmental defects. Another 5 patients received a VP-shunt postoperatively, all developmental defect cases with hydrocephalus.

No statistically significant differences were observed between groups for the analyzed variables (Table 1). There was a relatively high percentage of procedures with missing data for suture type, suture technique used, and attempt of watertight closure (Table 1).

### CSF leakage, associated complications, and treatment

CSF leakage occurred in 2.7% (2/75) of procedures. The first patient was a male newborn who underwent an untethering procedure. CSF leakage occurred on day 21. The patient was readmitted and underwent wound revision surgery 1 day later. The total readmission period was 7 days. The second patient was a 6-year-old boy who underwent a vascular procedure, and the leak occurred on day 3 after the index procedure. A pressure bandage was applied, and on day 4 after diagnosis of the leak, an external lumbar drain (ELD) was placed.

The ELD could be removed after 6 days and wound leakage did not recur. Hospital stay was prolonged with an estimated 10 days. The index procedure was the first surgery in both cases, and no dural grafts were used. The patients were not immunocompromised and had no hydrocephalus.

In total, 3 patients developed a pseudomeningocele or CSF leak (3.8%). There was one case of pseudomeningocele without a CSF fistula, which was treated with wound revision surgery. A total of 8 cases of SSI occurred (10.7%). Patients with a CSF leak had moderate evidence for an increased risk for SSI, where 6 of 73 (8.2%) patients without, and both patients with CSF leak developed an SSI, respectively (*p* = 0.01). One patient with an SSI (and no CSF leakage) was treated with surgical wound revision. All patients with SSI and CSF leakage received antibiotic treatment. There were no cases of meningitis in this cohort.

The CSF leakage risks in pediatric populations of intradural spinal surgery reported in the 4 studies identified in the literature range from 0 to 12.7% (Table 2).

**Table 2:** Literature overview of CSF leak after spinal surgery in the pediatric population

**Author**	**Year**	***N***	**CSF leak (%)**	**SSI (%)**	**Meningitis (%)**
Kaufman et al. [8]	2010	27	0.0	0.0	0.0
Liu et al. [9]^a^	2014	638	7.1	3.1	0.0
Goodwin et al. [7]	2014	93	5.4	NR	1.1
Balasubramaniam et al. [5]	2014	102	12.7	NR	NR
Slot et al. [3]^b^	2023	75	2.7	10.7	0.0

*CSF* cerebrospinal fluid, *N* number, *NR* not reported

^a^Percentage is based on the definition of CSF leakage including pseudomeningocele. The percentage of overt CSF leakage reported in this study is 2.8%

^b^Represents the current study

## Discussion

The incidence of CSF leakage, defined as leakage through the incision after spinal surgery in the pediatric population, was 2.7%. To our knowledge, this study is the first international, multicenter report of CSF leakage after intradural spinal surgery in children.

Definitions of CSF leakage in the current body of literature are not always described and may vary. Liu et al. reported a risk of 7.1%, which includes cases of PMC only as well. When looking at cases with incisional leakage in their large series of intradural spinal surgery specifically, they report 18/638 cases to have an overt CSF leakage (2.8%). This is comparable to the risk of incisional CSF leakage observed in our study (2.7%). The combined CSF leakage and PMC risk in our study is substantially lower: 3.8%. This discrepancy may be the result of the subjective definition of PMC, especially considering the retrospective nature of both studies. Another explanation may be the difference in the distribution of procedures performed between these studies. Kaufman et al. found no CSF leakage in their study reporting on the use of non-penetrating anastomotic clips to close the dura after spinal procedures [8]. This is however a small series, which includes several procedures with dural incisions of 10 mm or less in length [8]. The use of a PEG-hydrogel was evaluated in another study, which reported a 5.4% CSF leak risk in procedures in which this type of sealant was used [7]. Two studies investigating the association between the use of fibrin glue and CSF leakage did not find a statistically significant effect [5, 9]. Currently, we believe that there is insufficient evidence to recommend the use of additional methods to augment dural closure.

To achieve optimal watertight closure, the first choice is to close the dura primarily using microsurgical techniques. If this is not possible, the use of autologous duraplasty material is the second choice. The third choice would be to make use of an allogenic duraplasty. The low leakage rate observed in this spinal cohort is in line with the observation that for the largest group in this cohort, tumor resection cases, we generally find an intact dura to be opened surgically during the procedure, which we are able to close primarily, without the need for duraplasty. In the vast majority of cases, this involves laminotomy with the replacement of the lamina. For the other large group in our cohort, developmental disorders, we more often see a defect in the dura, for which a duraplasty has to be used. We advise to leave any subcutaneous lipoma in place for proper closure of the skin tissue to reduce the relatively higher leakage risk in these cases.

The CSF leakage risk found in this spinal cohort is lower than that found in the cohort of intradural cranial surgery procedures (7.5%) in the same centers for this time period [13]. This also applies when compared to most previously reported CSF leakage risks in intradural spinal surgery (0–12.7%) [5, 7, 9]. This contradicts the belief that the risk of CSF leakage after spinal surgery is higher due to increased intradural hydrostatic pressure in an upright position at the surgical site on the spinal level compared to cranially [9].

The percentage of surgical site infections in our series is relatively high which may partially be explained by our lenient definition of SSI (which does not require a positive wound culture). Previous studies report SSI risks between 0.3 and 12.7% [9, 14–17]. Liu et al. [9] find a significantly higher incidence of wound infection in patients with CSF leak compared to patients without in their study. However, they have also included PMC only in the definition of CSF leak. All cases of wound infection in that group, though, occurred in patients with an incisional leak, which confirms our rationale for considering PMC as a separate clinical entity. There were no cases of meningitis in our study, which is in line with the low incidence of meningitis reported in other publications [7, 9, 18].

Risk factors identified for CSF leakage in the study by Liu et al. after intradural spinal surgery in the pediatric population are previous spinal surgery, the use of a dural graft, older age, the use of non-locked continuous suturing, and the procedure performed [9]. Cord untethering operations had the highest CSF leak risk in their study. This is consistent with the relatively high CSF leak risk reported in the majority of other series including tethered cord syndrome procedures specifically [6, 10, 11, 14, 15, 17]. This can be explained by the inherent relation between the pathology in lipomyelomeningocele, often resulting in a dural defect at closure, and (myelo)meningocele and CSF leak [11]. Chern et al. find no association between CSF leak and surgery time, bed rest, use of a sealant, or use of the microscope in their retrospective series among patients who underwent cord untethering procedures for tight filum terminale [6]. One out of 2 CSF leakage cases in our study was an untethering procedure as well.

CSF leakage was treated with invasive treatment (ELD and revision surgery, respectively) in both cases in our study. Balasubramaniam et al. also report invasive treatment with lumbar drainage, placed a few millimeters from the incision, preferably one or two levels above, in all procedures and reoperation in 23% [5]. Liu et al. report a reoperation risk of 44.4% for incisional CSF leakage, whereas the majority of pseudomeningoceles resolved without any intervention [9]. In our series, one case of PMC without CSF leak was treated invasively.

The most important limitation of this study is the small sample size and the low number of CSF leaks (*N* = 2), which limits the validity of statistical analyses performed. Furthermore, this retrospective study is vulnerable to reporting bias as it depends on existing clinical records. The variables with missing data for suture type, suture technique used, and attempt of watertight closure reflect this bias. These variables could therefore not be incorporated into our statistical analyses. Furthermore, we did not collect data on bedrest prescription and level of the surgery. Yet, these factors may actually be of influence on the risk of CSF leakage and should thus be further evaluated in future studies.

The comparison to previous literature was limited by the unclear and varying definitions in the existing body of literature. We suggest to use the definition of CSF leakage as “leakage through the skin” and consider PMC separately in future studies.

This study shows that CSF leakage after intradural spinal surgery in the pediatric population is rare (2.7%). Nevertheless, the clinical consequences with respect to complications such as infection, necessity for invasive treatment, and prolonged hospitalization are serious. Future studies into the development of preventative strategies for spinal surgery in the pediatric population are warranted.

## Supplementary Information

Below is the link to the electronic supplementary material.

Supplementary file1 (DOCX 12 kb)

## Data Availability

The data that support the findings of this study are available from the corresponding author upon reasonable request.

## Abbreviations

CSF: Cerebrospinal fluid
ELD: External lumbar drainage
ETV: Endoscopic third ventriculostomy
EVD: External ventricular drainage
IQR: Interquartile range
PICU: Pediatric intensive care unit
PMC: Pseudomeningocele
SSI: Surgical site infection
VP: Ventriculoperitoneal
