# Supplementary material for: Cerebrospinal fluid leakage after intradural spinal surgery in children
Source: Childs Nerv Syst. 2023 Feb 15;39(4):1013–9. doi: 10.1007/s00381-022-05797-w (PMC10160141; doi:10.1007/s00381-022-05797-w)
Supplement: Supplementary file 1 — Supplementary file1 (DOCX 12 kb) [file 381_2022_5797_MOESM1_ESM.docx]

**Supplemental table 1.** Inclusion and exclusion criteria

| **Inclusion criteria** |
| --- |
| Intradural spinal surgery |
| ≤18 years old at time of surgery |
| Surgical report available |
| **Exclusion criteria** |
| Death within 6 weeks after surgery |
| Lost to follow-up within 6 weeks after surgery |
| Reoperation for other reason than CSF leakage within 6 weeks after surgery |
